# Inhibitory mechanism of quercetin on *Alicyclobacillus acidoterrestris*

**DOI:** 10.3389/fmicb.2023.1286187

**Published:** 2023-11-15

**Authors:** Xinhong Liang, Cunjian Tu, Yongchao Li, Junliang Sun, Ruixiang Zhao, Junjian Ran, Lingxia Jiao, Junchao Huang, Junrui Li

**Affiliations:** ^1^School of Food Science, Henan Institute of Science and Technology, Xinxiang, China; ^2^School of Life Sciences, Henan Institute of Science and Technology, Xinxiang, China

**Keywords:** quercetin, *Alicyclobacillus acidoterrestris*, antibacterial mechanism, juice, cell membrane

## Abstract

In this the antibacterial of quercetin against *Alicyclobacillus acidoterrestris* was evaluated by measuring the minimal inhibitory concentration (MIC) and minimal bactericidal concentration (MBC). Subsequently, the effect of quercetin on *A. acidoterrestris* cell membrane was evaluated through scanning electron microscopy (SEM), surface hydrophobicity determination, diacetate fluorescein staining and propidium iodide (PI) staining. Additionally, the effects of quercetin on intracellular macromolecules and cell metabolism were explored by measuring the culture medium protein, bacterial protein and intracellular sodium and potassium adenosine triphosphate (ATP) enzyme activity. The results revealed that quercetin exhibited the MIC and MBC values of 100 ug/mL and 400 ug/mL, respectively, against *A. acidoterrestris*. The SEM results revealed that quercetin could induce irreversible damage to the cell membrane effectively. Moreover, quercetin could enhance the surface hydrophobicity of *A. acidoterrestris*. The results of flow cytometry and fluorescence microscopy analyses revealed that quercetin could promote cell damage by altering the cell membrane permeability of *A. acidoterrestris*, inducing the release of nucleic acid substances from the cells. Furthermore, the determination of protein content in the culture medium, bacterial protein content, and the Na(+)/K(+)-ATPase activity demonstrated that quercetin could reduce the intracellular protein content and impedes protein expression and ATPase synthesis effectively, leading to apoptosis.

## 1. Introduction

*Alicyclobacillus acidoterrestris* is an acidophilic, heat-resistant, Gram-positive, non-pathogenic bacterium widely distributed in soil or acidic juices. It could thrive in a temperature range of 26–60°C, with an optimal temperature ranging between 42–53°C, and its growth pH spans from 2.0 to 6.0, with an optimum pH ranging between 3.5 and 5.0 (Smit et al., 2011). After being cultured liquid medium for 4–6 h, the cells enter the logarithmic growth phase, while spore formation occurs after 48 h. It is reported that both *A. acidoterrestris* and its spores have the potential to contaminate the intermediate products and production materials used in fruit juice processing, encompassing fruit juices and processed water. Therefore, detecting the presence of *A. acidoterrestris* and its spores at the initial stages of contamination is challenging. Under favorable external conditions, *A. acidoterrestris* germinates and develops into vegetative cells, leading to the precipitation of fruit juice products, thereby producing an unpleasant odor and reducing the commercial value (Chang and Kang, 2004; Bevilacqua et al., 2009).

So far, many sterilization methods have been employed by the juice processing industries to ensure microorganism safety. Among them, heat pasteurization can trigger the germination of Alicyclobacillus spores better. Although non-thermal pasteurization techniques like high hydrostatic pressure, supercritical carbon dioxide, and ultraviolet radiation, have the potential to serve as the alternatives, the application of some methods are constrained due to their expensiveness (Ashrafudoulla et al., 2023). In contrast, eco-friendly and safe natural antibacterial compounds offer cost-effective, user-friendly solutions, effectively suppressing bacterial growth during juice storage. As such, these compounds have gained extensive adoption in the juice industry (Yamazaki et al., 2000; Bevilacqua et al., 2014). Molva and Baysal (2015a) investigated the antibacterial effect of grape seed extract on *A. acidoterrestris* and found that this compound could inhibit the growth of vegetative cells and spores *A. acidoterrestris*. Molva and Baysal (2015b) inoculated *A. acidoterrestris* into sterile apple juice infused with pomegranate extract, adjusting the bacterial concentration to 10^5^ CFU/mL. After 240 h of cultivation, the bacterial count in the experimental group was reduced to approximately 10^3^ CFU/mL. The SEM results displayed significant damage to the bacterial cell walls and membranes, with some cells exhibiting breakage or dissolution.

Quercetin is a natural flavonoid, widely distributed in nature, such as tea, coffee, honeysuckle, and onions (Zou et al., 2021). Herbal and plant remedies continue to attract attention as future antibacterial therapies (Mevo et al., 2021). Studies have shown that quercetin and its derivatives have excellent antibacterial (Nguyen and Bhattacharya, 2022), antioxidation (Fuentes et al., 2017), anti-inflammatory (Lesjak et al., 2018), anticancer (Reyes-Farias and Carrasco-Pozo, 2019), antivirus (Saakre et al., 2021), and hypoglycemic properties (Wei et al., 2021). Quercetin have been widely used in medicine, health care, food and other fields (Patel et al., 2018). It is reported that quercetin exhibits a distinct antibacterial effect against both Gram-positive and Gram-negative bacteria, including *Staphylococcus aureus*, *Escherichia coli*, and *Pseudomonas aeruginosa* (Amin et al., 2015; Ouyang et al., 2016; Sarangapani and Jayachitra, 2018; Mu et al., 2021). Jaisinghani (2017) investigated the antibacterial properties of quercetin against *P. aeruginosa* and *E. coli* and found that quercetin exhibited the best inhibitory impact on these bacteria at concentrations of 300 μg/mL and 400 μg/mL, respectively. Kim et al. (2022) investigated the ability of quercetin to impede the formation of Salmonella biofilms, indicating its potential as a biofilm inhibitor.

Although some studies have examined the antibacterial effects of quercetin on various bacteria, the role of quercetin in preventing the growth of *A. acidoterrestris* has not been fully assessed yet. Therefore, the present study aimed to evaluate the antibacterial activity of quercetin against *A. acidoterrestris*. Additionally, the effect of quercetin cell membrane permeability was investigated through SEM, surface hydrophobicity assessment, fluorescein diacetate (FDA) staining, and PI staining. Furthermore, the protein content in the culture medium, bacterial protein content, and intracellular ATPase activity were assessed. Overall, the experimental results provide insights into the antibacterial mechanism of quercetin against *A. acidoterrestris*, laying a foundation for developing innovative strategies for the juice processing industry to mitigate microbial contamination effectively.

## 2. Materials and methods

### 2.1. Materials and chemicals

Quercetin was purchased from Sigma-Aldrich Shanghai Trading Co., Ltd. (Shanghai, China). aTPase (Na +, K +) test box Suzhou Grius Biotechnology Co., Ltd. (Suzhou, China). *A. acidoterrestris* DSM 3922^T^ was purchased from Deutsche Sammlung von Mikroorganismen und Zellkulturen (DSMZ, Braunschweig, Germany).

### 2.2. Activation and culture conditions of *Alicyclobacillus acidoterrestris*

The frozen strains were thawed at room temperature, and the activated bacterial solution was coated onto the AAM medium plate and incubated at 45°C for 24 h. Then, a single colony was selected from the plate and inoculated into 50 mL of AAM liquid culture medium. It was incubated at 45°C and 250 r/min for 12 h. Subsequently, the bacterial solution was transferred to 100 mL of AAM liquid culture medium with a 1.0% concentration. The culture was halted at the logarithmic phase and stored in a refrigerator at 4°C for future use.

### 2.3. Determination of MIC and MBC of quercetin on *Alicyclobacillus acidoterrestris*

The MIC was assessed following the procedure outlined by Bączek et al. (2017). Quercetin was diluted in the medium to yield concentrations of 800, 400, 200, 100, 50, 25, 12.5, 6.25, 3.12, and 1.56 mg/mL. Both the liquid medium and a blank control without bacteria were exclusively supplemented with quercetin, designated as CK_1_ and CK_2_, respectively. Following thorough mixing, 100 μL of AAM liquid medium was subjected to a 10^3^-fold dilution. Subsequently, the diluted samples were cultured in incubators at 45°C for 24 h, with observation directed towards tube transparency and clarity. The minimal concentration demonstrating no turbidity was identified as the MIC. Based on the determined MIC, the culture medium from the 100 μL clear test tube was uniformly spread onto the corresponding solid medium. This process was iterated three times. Viable bacteria presence was examined in the incubator at 45°C for 24 h, ultimately determining the lowest concentration without observable viable bacteria – designated as the MBC.

### 2.4. Effect of quercetin on cell morphology of *Alicyclobacillus acidoterrestris*

The logarithmic phase of *A. acidoterrestris* was added to quercetin with a final concentration of 1/2 MIC, 1 MIC, and 2 MIC, and cultured in a shaker at 250 r/min, 45°C for 6 h. Taked 1 mL of the liquid to be tested in a 2 mL centrifuge tube, centrifuged at 10000 r/min for 5 min, discarded the supernatant, rinsed with pre-cooled sterile water for 2–3 times, leaving only the layer precipitate, and fully resuspended with 1 mL 2.5% pentanediol to achieve the purpose of fixing the cell morphology. The sample was refrigerated at 4°C for over 2 h. Following centrifugation, the supernatant was removed, and the residue was washed with sterile water before undergoing an additional 1-min centrifugation. Once again, the supernatant was discarded, and 1 mL of corresponding alcohol concentration was introduced. Placing the solution in a dark environment within a 4°C refrigerator for 10–15 min followed, after which the supernatant was eliminated via centrifugation. For analysis, 10 μL of the diluted bacterial solution was applied to the prepared small discs, employing the tip of a pipette gun. Subsequent drying was conducted, and cell morphology was examined by a scanning electron microscope (SS550, Carl Zeiss, Germany) (Chen et al., 2017).

### 2.5. Effect of quercetin on surface hydrophobicity of *Alicyclobacillus acidoterrestris*

Cell hydrophobicity was determined by the methods of Kimoto-Nira et al. (2010) with some modifications. *A. acidoterrestris* culture (30 mL) was subjected to centrifugation at 8000 r/min for 10 min within a 50 mL centrifuge tube. Following the removal of the supernatant, two washes with 0.01 M PBS buffer were performed. Subsequently, the bacterial concentration was fine-tuned to 10^8^ CFU/mL.3 mL of each bacterial solution was taken, and 200 μL of 2 MIC honeysuckle leaf extract solution was added for 0, 15, 30, 45, and 60 min, respectively. After the action, 1.6 mL of CHCl_3_ was added. The vortex instrument was used to vortex for 30 s and then stood at room temperature for 30 min. After the liquid was stratified, the supernatant was removed by a pipette gun. The absorbance of the liquid to be measured at 600 nm wavelength was measured by a visible light spectrophotometer (7,200, Unico, China). Each sample solution was set up in parallel 3 groups. The calculation formula of cell adsorption rate is as follows:


(1) $$\mathrm{cell}\;\mathrm{absorption}\;\mathrm{rate}=\frac{\mathrm{blank}\;\mathrm{control}\;\mathrm{group}-\mathrm{sampling}\;\mathrm{group}}{\mathrm{blank}\;\mathrm{control}\;\mathrm{group}}\times 100\%$$

### 2.6. Effect of quercetin on cell membrane permeability of *Alicyclobacillus acidoterrestris*

The assessment of cell membrane permeability followed the procedure outlined by Sánchez et al. (2010). Following the logarithmic growth phase, the culture underwent centrifugation at 6000 r/min at 4°C for 10 min. Subsequent to discarding the supernatant, a resuspension in PBS took place. The bacterial concentration was regulated to approximately 10^7^ CFU/mL. Then, varying concentrations of quercetin (1/2 MIC, 1 MIC, and 2 MIC) were introduced, along with a PBS-based blank control. The cells were cultured in a shaker at 250 r/min and 45°C for 6 h. Subsequently, the bacterial suspension from each group underwent centrifugation at 6000 r/min and 4°C for 10 min to gather the cells. After undergoing two washes with PBS, the supernatant was eliminated via centrifugation, followed by the addition of 250 μL of a FDA-acetone solution (2 mg/mL). Following a 20-min incubation at room temperature, the samples were subjected to three washes using PBS. Subsequent to centrifugation at 8000 r/min and 4°C for 10 min, the supernatant was discarded, and the cells were resuspended in PBS. fluorescence intensity was quantified using a fluorescence spectrophotometer (G 9800A, Agilent, United States) with an excitation wavelength set at 297 nm and an emission wavelength set at 527 nm.

### 2.7. Effect of quercetin on membrane damage of *Alicyclobacillus acidoterrestris*

The method outlined by Yadav et al. (2018) was referenced with minor adjustments. A total of 4.5 mL of *A. acidoterrestris* bacterial solution in the logarithmic phase was drawn into a conical flask. Within these samples, each of the three groups received 0.5 mL of quercetin, each at a concentration of 1/2 MIC, 1 MIC, and 2 MIC, respectively. In contrast, the remaining group received 0.5 mL of AAM medium and was designated as the blank control. The culture was incubated in a culture box at 45°C and 250 r/min for 6 h. Afterward, 1 mL of the treated sample solution was withdrawn and subjected to centrifugation at 8000 × g for 5 min, resulting in the removal of the supernatant. Subsequently, 1 mL of PBS buffer was introduced, and this washing process was repeated thrice. Ultimately, the bacterial suspension was resuspended in PBS buffer, yielding a final concentration of approximately 10^6^ CFU/mL. A 1 mL portion of the sample solution was collected into a 2 mL centrifuge tube, followed by centrifugation at 6000 × g for 10 min. Subsequently, the supernatant was discarded, and 1 mL of PBS buffer solution was introduced to achieve complete resuspension. Following resuspension, 5 μL of PI dye was added. After thorough mixing, the solution was placed in the refrigerator at 4°C for 30 min in a dark environment. Subsequent to the incubation period, the mixture was removed and subjected to centrifugation at 5000 r/min for 5 min. Finally, resuspension with PBS buffer was conducted for subsequent use.

Fluorescence inverted microscope (A1FL-LED, Carl Zeiss, Germany) was utilized to visualize the staining of *A. acidoterrestris* post quercetin treatment. Specifically, 5 μL of the target bacteria was applied to coat a cover glass on a slide, which was subsequently positioned onto a fluorescence microscope stage. Employing a 100-fold oil objective, the red fluorescence intensity emitted by the bacteria was examined to assess the extent of damage to the *A. acidoterrestris* membrane.

*Alicyclobacillus acidoterrestris* following quercetin treatment was assessed using flow cytometry (CytoFLEX, Beckman Coulter, United States). The sample solution underwent a 5-min ultrasonic treatment and was subsequently filtered through a membrane filter. This process aided in evenly dispersing cells of higher concentration, thereby facilitating observation via flow cytometry. Subsequently, fluorescence measurements were recorded using the machine, with channel selection set to FL2-H::PE-H. The acquired results were analyzed by Flowjo software.

### 2.8. Effect of quercetin on the content of bacterial protein and culture medium protein of *Alicyclobacillus acidoterrestris*

The extraction method of bacterial protein was based on the report of dos Anjos et al. (2016). Four groups of 9 mL PBS buffer were set up, and 100 μL of *A. acidoterrestris* in logarithmic phase was added to each group of test tubes. Three groups of them were added with 1 mL different concentrations of quercetin (concentrations were 1/2 MIC, 1 MIC, 2 MIC), and the other group was added with 1 mL medium as a blank group control, placed at 250 r/min, 45°C cultured for 6 h. After 6 h of culturing, the cells were centrifuged at 6000 r/min at 4°C for 10 min. The supernatant was collected, and the protein concentration of the culture medium was determined using a microplate reader (Varioskan Flash 3,001, Thermo, United States). The cells were resuspended in 0.01 M PBS to achieve a concentration of approximately 10^6^ CFU/mL. An equivalent volume of bacterial suspension was taken and subjected to disruption in an ultrasonic cell disruptor (VCX500, Sonics, United States) for 20 min. Following this, centrifugation was conducted at 6000 r/min at 4°C for 10 min. The resulting supernatant was collected, and the concentration of bacterial protein was quantified using a microplate reader.

The supernatant was used to determine the concentration of bacterial protein by microplate reader, The standard regression equation of relative protein concentration calculated by Coomassie brilliant blue G-250 staining method (y = 0.003x + 0.2805, *R*^2^ = 0.9984), x is protein concentration, y is absorbance. According to the above equation, the content of protein in the sample was determined.

### 2.9. Effect of quercetin on ATPase activity of *Alicyclobacillus acidoterrestris*

The *A. acidoterrestris* cultured to the logarithmic growth phase (10^7^ CFU/mL) was centrifuged at 8000 r/min for 10 min at 4°C, and the supernatant was discarded. The bacterial cells were suspended in sterile PBS (pH 7.0), undergoing repeated cycles of centrifugation and washing three times. Ultimately, the cells were resuspended in an equal volume of PBS. Quercetin was subsequently introduced at final concentrations of 1/2 MIC, 1 MIC, and 2 MIC, respectively, with sterile deionized water serving as a blank control. The supernatant was discarded by centrifugation to gather the precipitated bacteria. Subsequently, the centrifuged bacteria were combined with 1 mL of extract, and the bacterial cells were disrupted in an ice water bath by an ultrasonic disruptor (VCX500, Sonics, United States). Each ultrasonic cycle lasted for 3 s, with a 10 s interval, and this procedure was repeated 30 times. Afterward, centrifugation was carried out at 12,000 rpm at 4°C for 10 min. The resulting supernatant was collected and stored on ice for subsequent testing. The activity of Na(+)/K(+)-ATPase in *A. acidoterrestris* cells was determined using a Na(+)/K(+)-ATPase kit at 700 nm.

### 2.10. Statistical analysis

The results were expressed by the mean ± standard deviation, the samples were replicated three times, the data were analyzed by SPSS 23.0 software, and the analysis of variance was calculated by the q-test multiple comparison method. *p* < 0.05 indicated that there was a significant difference in the statistics.

## 3. Results

### 3.1. Determination of MIC and MBC of quercetin against *Alicyclobacillus acidoterrestris*

**Figure 1:**
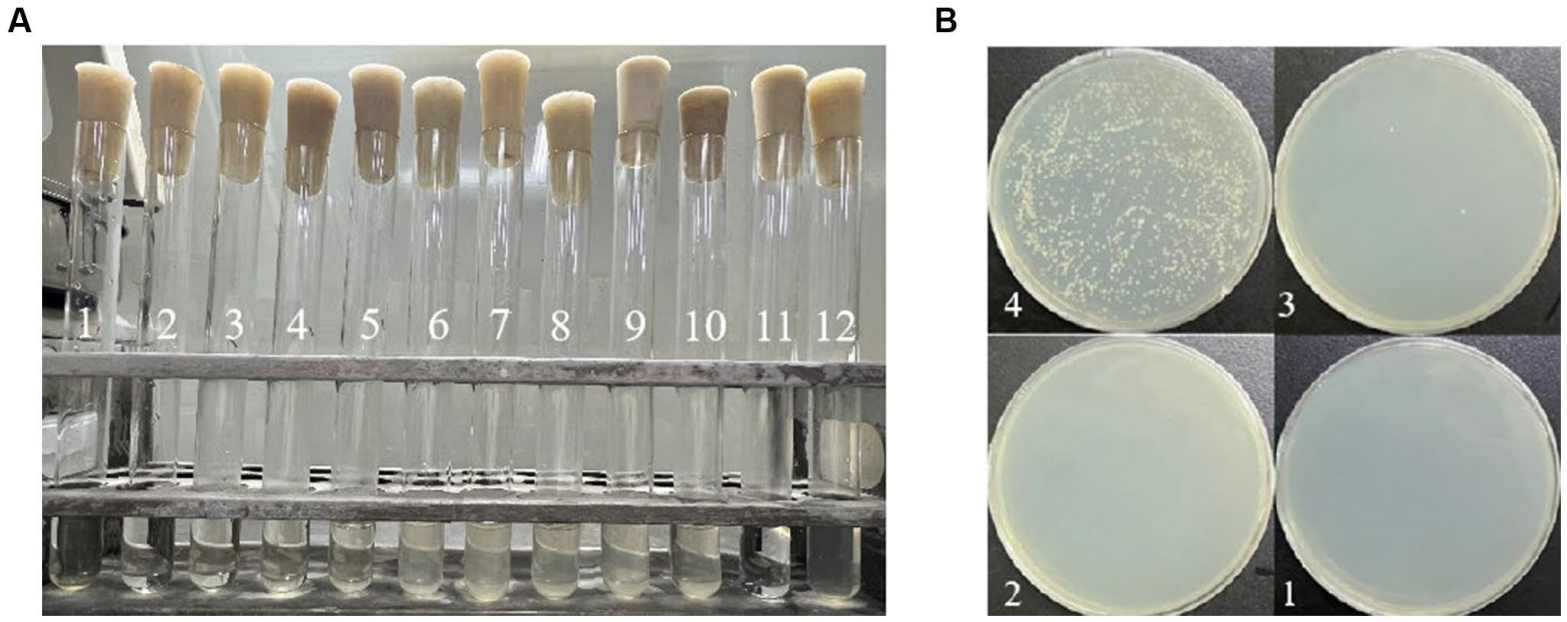
Determination of MIC and MBC of quercetin on *A. acidoterrestris*: **(A)** MIC determination results; the content of quercetin was 800 mg/mL in No. 1 tube, 400 mg/mL in No. 2 tube, 200 mg/mL in No. 3 tube, 100 mg/ mL in No. 4 tube, 50 mg/mL in No. 5 tube, 25 mg/mL in No. 6 tube, 12.5 mg/mL in No. 7 tube, 6.25 mg/mL in No. 8 tube, 3.12 mg/mL in No. 9 tube, and 1.56 mg/mL in No. 10 tube. No. 11 tube was blank control tube and No. 12 tube was negative control tube. **(B)** MBC determination results; the quercetin content of No. 1 plate was 800 mg/mL, the quercetin content of No. 2 plate was 400 mg/mL, the quercetin content of No. 3 plate was 200 mg/mL, and the quercetin content of No. 4 plate was 100 mg/mL.

The results of MIC and MBC are depicted in Figure 1. In Figure 1A, tube 11 represents the blank control, exhibiting clarity and signifying the absence of bacterial contamination due to quercetin. Conversely, tube 12, the negative control, appears turbid, indicating the suitability of the medium for *A. acidoterrestris* growth. Tubes 1 to 4 exhibited clarity, while tube 5 began to show turbidity. Thus, the MIC of quercetin against the tested bacterium *A. acidoterrestris* was observed at the point of 4 tubes, equivalent to 100 ug/mL. Subsequently, 100 μL of the liquid from test tubes 1 to 4 was uniformly spread on the agar plate, and the results are depicted in Figure 1B. Notably, bacterial growth was absent in No. 1 and No. 2, with consistent results across repeated tests. As a result, No. 2 was identified as the MBC, corresponding to *a* value of 400 ug/mL.

### 3.2. Effect of quercetin on cell morphology of *Alicyclobacillus acidoterrestris*

**Figure 2:**
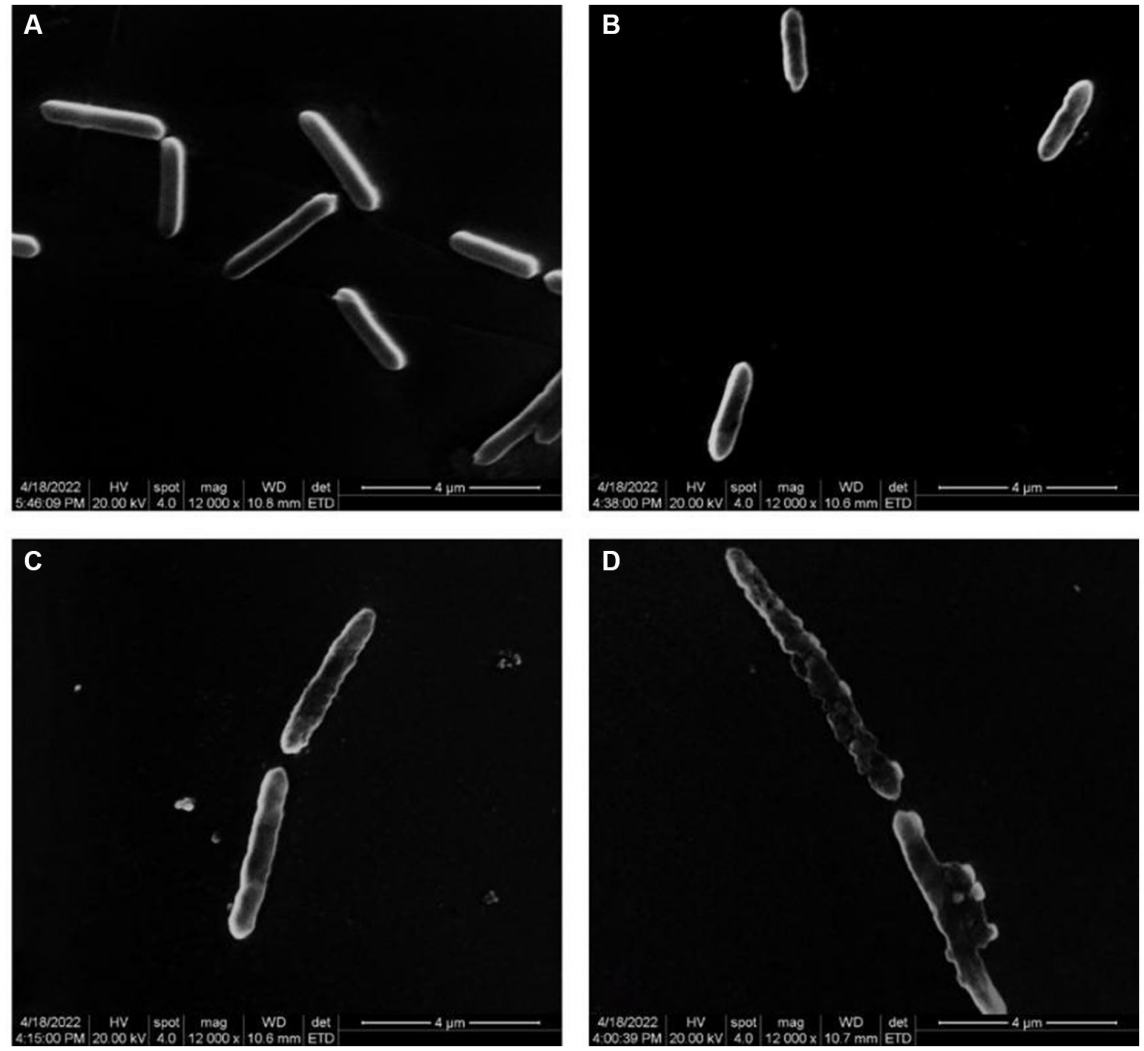
Electron microscope images of different concentrations of quercetin treatment on *A. acidoterrestris*. **(A)** Control, **(B)** 1/2 MIC, **(C)** 1 MIC, and **(D)** 2 MIC.

Figure 2 illustrates the impact of varying quercetin concentrations on the morphology of *A. acidoterrestris*. When *A. acidoterrestris* was cultured to the logarithmic phase without quercetin, the bacteria exhibited an intact, rod-shaped morphology with a smooth surface devoid of cell damage or content overflow. When 1/2 MIC of quercetin was added and cultured for 6-h, *A. acidoterrestris* showed noticeable changes, such as a wrinkled appearance, with a rough surface lacking the characteristic smoothness and flatness. Additionally, evident depressions were observed in certain areas, indicating a significant impact on bacterial growth. At a quercetin concentration of 1 MIC, *A. acidoterrestris* exhibited significant damage. Small holes appeared on the surface, resulting in an uneven and irregular shape. The cell matrix displayed a phenomenon of leakage, causing substantial harm to the bacteria. These observations indicated an abnormal growth pattern of the bacteria at this concentration. In contrast, the cells exposed to a quercetin concentration of 2 MIC demonstrated severe damage, characterized by nearly complete cell membrane rupture. The cells became transparent, and their contents dissolved, signifying an apoptotic process occurring at 2 MIC. Furthermore, certain cells displayed elongation across multiple cell sizes, which might be attributed to the growth-inhibiting impact of quercetin on *A. acidoterrestris* that, disrupted the external cell structure, leading to the release of proteins, DNA, and RNA. Molva and Baysal (2015b) observed the morphological changes of *A. acidoterrestris* cells treated with different concentrations of pomegranate fruit extracts by SEM, and found that the cells in the control group had complete cell walls and smooth surface cell membranes. On the contrary, the cells treated with pomegranate fruit extract showed ruptured cell wall and cell membranes. As the concentration increased, the deformation of *A. acidoterrestris* became more serious, and some of the cells were broken or even dissolved, indicating that the *A. acidoterrestris* cells treated with pomegranate fruit extract had serious morphological damage, which was consistent with the results of this study.

### 3.3. Effect of quercetin on the surface hydrophobicity of *Alicyclobacillus acidoterrestris*

**Figure 3:**
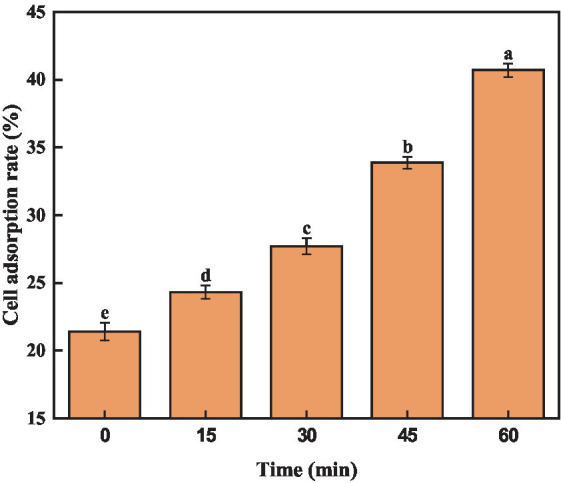
Effect of quercetin on surface hydrophobicity of *A. acidoterrestris*. The same letter of a–e indicates no significant difference (*p* > 0.05), while different letters indicate significant difference (*p* < 0.05).

Previous reports have demonstrated a positive correlation between cell surface hydrophobicity and adhesion capability, which serves as an indicator of cell membrane alterations (Del Re et al., 2000). Trichloromethane, being hydrophobic, not harmful to bacteria. Consequently, assessing the adsorption rate of trichloromethane to *A. acidoterrestris* cells could indicate the alteration in surface hydrophobicity. As depicted in Figure 3, quercetin treatment notably elevated the cell surface hydrophobicity of *A. acidoterrestris*, leading to a gradual increase in chloroform adsorption over time. The adsorption rate of chloroform by *A. acidoterrestris* increased from 21.40% in the control group to 24.32%, 27.70%, 33.87%, and 40.70%, respectively. The adsorption rate approximately doubled after 60 min of treatment. These findings indicated that quercetin treatment significantly altered the surface properties of *A. acidoterrestris*, thereby increasing the cell surface hydrophobicity. This change might be attributed to the adsorption of quercetin onto the surface of the negatively charged cell membrane through electrostatic interactions. Such interactions inflict substantial damage to the cell membrane surface, thus exposing the hydrophobic regions of the membrane. This phenomenon significantly contributes to the observed increase in the cell surface hydrophobicity. Kong et al. (2008) found that quercetin could act on the bacterial cell membrane and destroy the cytoplasmic membrane structure due to its hydrophobicity, thereby inhibiting bacterial growth.

### 3.4. Effect of quercetin on cell membrane permeability of *Alicyclobacillus acidoterrestris*

**Figure 4:**
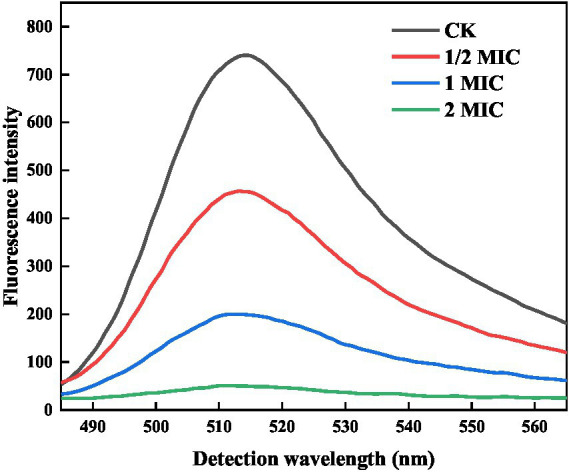
Effect of quercetin on FDA fluorescence intensity of *A. acidoterrestris*.

FDA is a non-fluorescent, uncharged lipid molecule that can penetrate the cell easily through its membrane. It can undergo hydrolysis by cellular enzymes, resulting in fluorescence emission. When the cell membrane is compromised, fluorescein is rapidly released, leading to a reduction in FDA fluorescence intensity. As a result, the permeability of the bacterial cell membrane can be determined through FDA fluorescence intensity (Sánchez et al., 2010). As shown in Figure 4, the fluorescence intensity of the control group was 740 a.u., suggesting that the cell membrane structure was relatively intact, and the permeability changes were minimal. Comparatively, the quercetin group exhibited significantly lower fluorescence intensity than the control group. Additionally, the fluorescence intensities at 1/2 MIC, 1 MIC, and 2 MIC were 456, 199, and 50 a.u., respectively. Notably, within these, the 2 MIC group demonstrated the most pronounced decrease in fluorescence intensity. These results demonstrated that the control group could not alter the permeability of the bacterial cell membrane. However, as the quercetin concentration increased, the extent of damage to the bacterial cell membrane became progressively more evident. Notably, the 2 MIC treatment group exhibited the lowest fluorescence intensity, signifying that the introduction of quercetin at a concentration of 2 MIC could significantly break the cell membrane of *A. acidoterrestris*, thereby increasing the cell membrane permeability. This observation was consistent with the findings of Figure 2, wherein the cell morphology of the 2 MIC group was markedly compromised, and the cell membrane was virtually disrupted.

### 3.5. Effect of quercetin on membrane damage of *Alicyclobacillus acidoterrestris*

**Figure 5:**
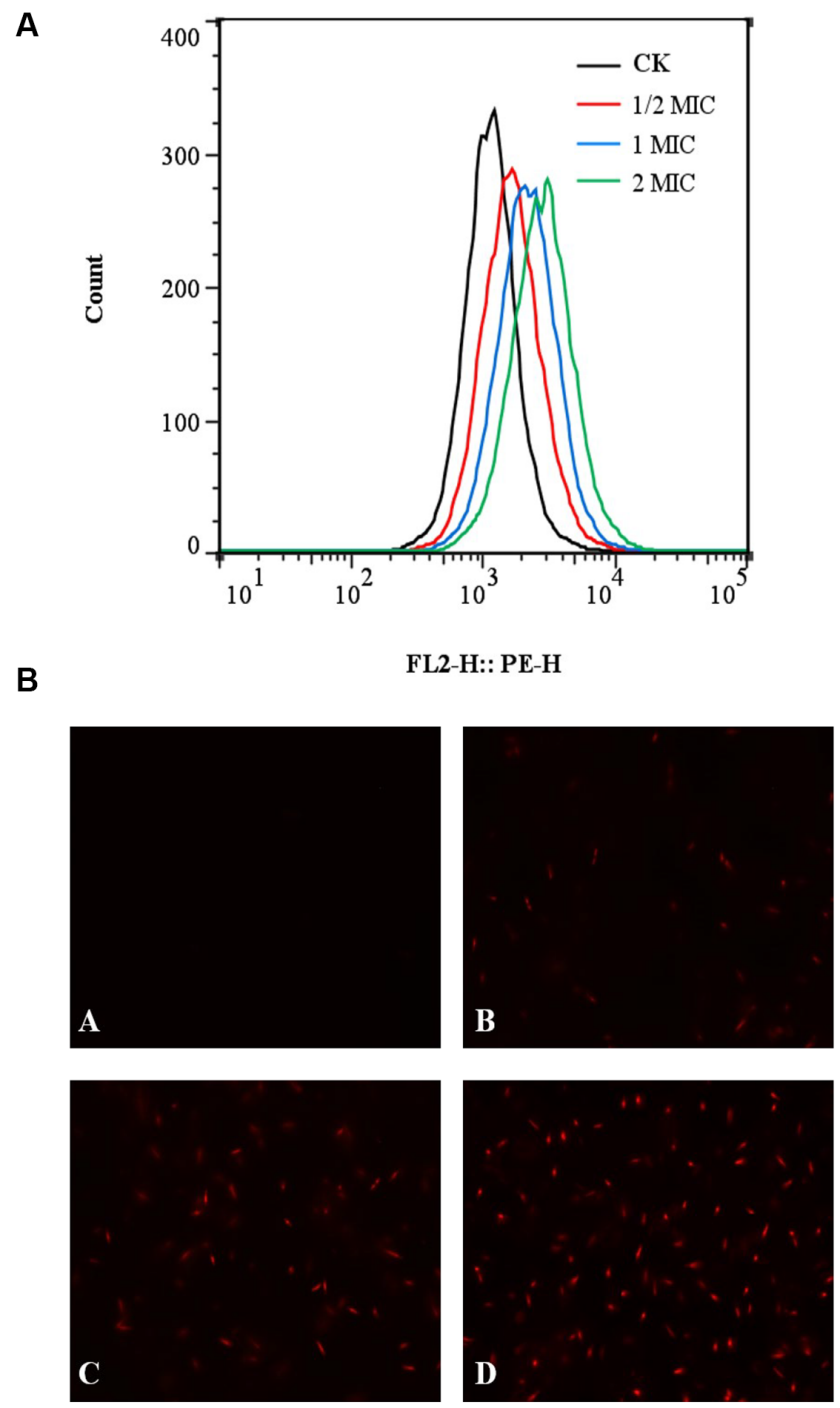
Effect of quercetin treatment on membrane damage of *A. acidoterrestris*. **(A)** Representative analysis diagram of flow cytometry and **(B)** Fluorescence microscope diagram, (A) Control, (B) 1/2 MIC, (C) 1 MIC, and (D) 2 MIC.

The fluorescent dye PI was applied to stain the cells both before and after quercetin treatment to assess the impact of quercetin on *A. acidoterrestris* cell membranes. Subsequently, flow cytometry was employed to identify and quantify any cell membrane damage. PI dye functions as a nuclear stain, capable of permeating a compromised cell membrane and reddening the nucleus, resulting in the generation of red fluorescence. The extent of red fluorescence indicates the number of cell deaths (Martins et al., 2018; Xu et al., 2018). The results of flow cytometry are shown in Figure 5A. Compared with the control group, the fluorescence peaks collected by flow cytometry significantly shifted to the right in the quercetin-treated group. When the concentration of quercetin was 2 MIC, the main peak shifted to the right, indicating that PI combined with the nucleic acid substances, and the cell membrane of *A. acidoterrestris* treated with quercetin changed. These findings were consistent with the fluorescence microscopy results. The outcomes of fluorescence inverted microscopy are depicted in Figure 5B. As shown in Figure 5B, no staining was observed in the control group, signifying the intact nature of the cell membranes within this group. Conversely, *A. acidoterrestris* treated with quercetin exhibited significant red fluorescence. Notably, the extent of red fluorescence expanded with quercetin concentration. This trend suggested that quercetin facilitated cell damage by inducing alterations in the cell membrane permeability of *A. acidoterrestris*. Consequently, this alteration led to the efflux of nucleic acid substances within the cells, which were subsequently stained red by the PI fluorescent dye. This effect became more pronounced with higher quercetin concentrations, highlighting the impact on cell membrane permeability further.

Fan et al. (2021) investigated the impact of thymoquinone on the membrane integrity of *A. acidoterrestris* using laser scanning confocal microscopy and fluorescent dye PI and found negligible red fluorescence in the control group. However, upon treatment with 1 MIC thymoquinone, a modest amount of red fluorescence was observed, indicating the disruption of membrane integrity in certain cells. Notably, at a concentration of 2 MIC, a substantial rise in red fluorescence intensity was observed, implying the significant capacity of thymoquinone for inducing severe cell membrane damage. Pal and Tripathi (2020) studied the impact of quercetin on the viability of *E. coli* cells and found that quercetin could significantly inhibit the viability of *E. coli* cells. Moreover, as the quercetin concentration increased, a corresponding increase in the number of red fluorescence signals was observed, indicating the increased permeability of *E. coli* cell membrane, which was consistent with the present study results.

### 3.6. Effect of quercetin on the content of bacterial protein and culture medium protein of *Alicyclobacillus acidoterrestris*

**Figure 6:**
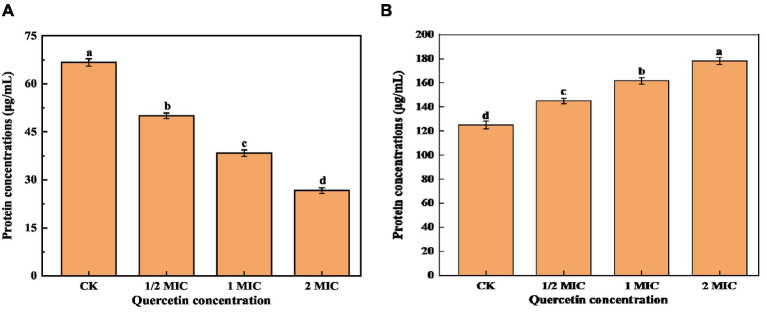
Effect of quercetin on the content of bacterial protein and culture medium protein of *A. acidoterrestris*. **(A)** The effect of quercetin on the bacterial protein of *A. acidoterrestris*. **(B)** Effect of quercetin on the protein of *A. acidoterrestris* culture solution, the same letter a–d indicates no significant difference (*p* > 0.05), while different letters indicate significant difference (*p* < 0.05).

Proteins are the foundational material of life and constitute a vital element within the cell body. They are intricately connected to numerous physiological and metabolic functions within the cells. Therefore, the disruption of the cell membrane can result in the leakage of intracellular proteins (Yao et al., 2014; Yin et al., 2020). As shown in Figure 6A, the intracellular protein concentration was significantly lower than that of the control group. With the increase in quercetin treatment concentration, the intracellular protein content decreased by 25% at the lowest concentration and 60% at the highest concentration compared to the control group. This indicates that quercetin caused increased permeability or cell membrane damage in *A. acidoterrestris*, leading to the leakage of macromolecular substances such as proteins from the cell membrane into the culture medium. As shown in Figure 6B, the protein concentration in the culture medium treated with quercetin increased significantly. With the increase in concentration, the protein content in the culture medium gradually increased from 125.26 μg/mL to 178.33 μg/mL. This result suggested that quercetin severely damaged the cell membrane, leading to substantial protein leakage, which was consistent with the electron microscopy findings. The loss of intracellular protein could be attributed to the interaction between the hydroxyl group of quercetin and the cell membrane through hydrogen bonds, which damaged the membrane structure and enhanced cell membrane permeability (Meng et al., 2016). Cai et al. (2019) investigated the impact of various concentrations of thymol on the protein content of *A. acidoterrestris*. As the concentration of thymol increased, the extent of protein leakage from *A. acidoterrestris* cells increased, indicating that thymol led to the destruction of *A. acidoterrestris* cells, causing the leakage of nucleic acids and proteins.

### 3.7. Effect of quercetin on ATPase activity of *Alicyclobacillus acidoterrestris*

**Figure 7:**
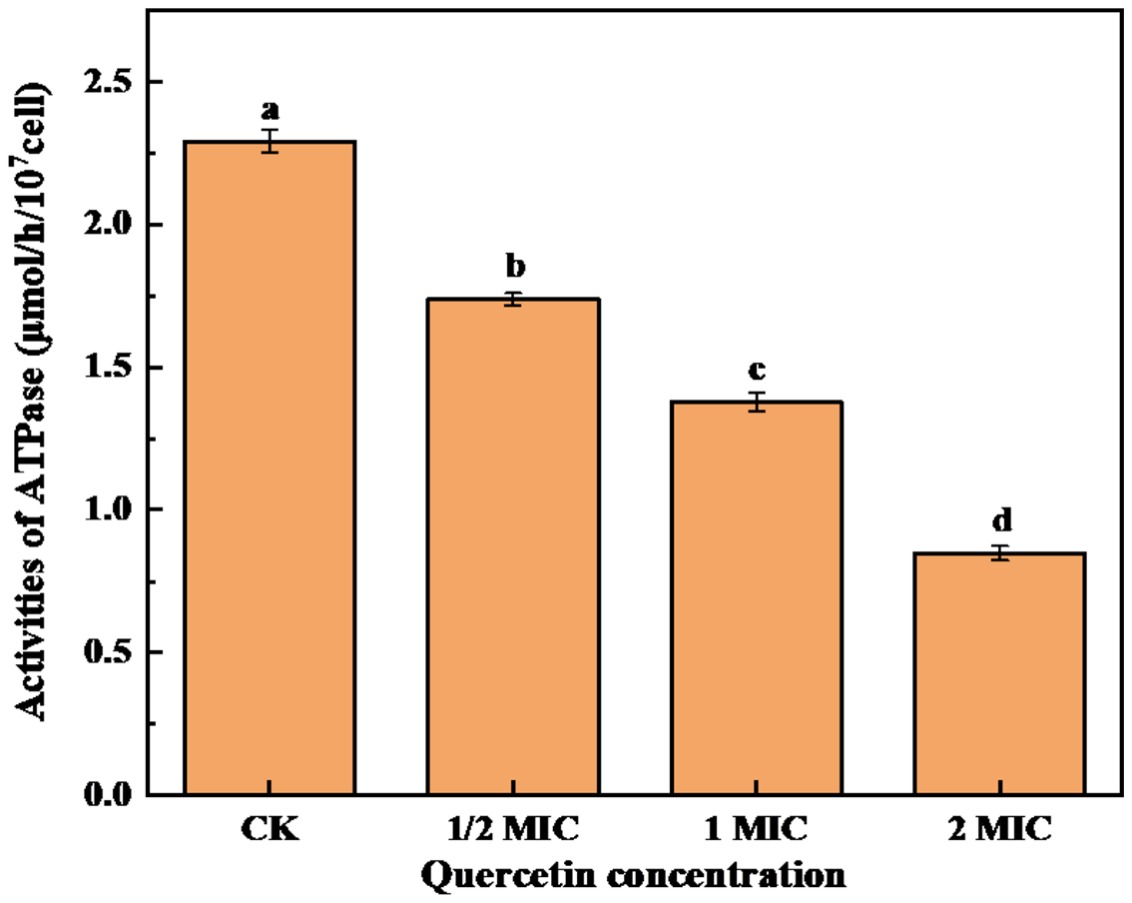
Effect of quercetin treatment on ATPase activity of *A. acidoterrestris* (a–d). The same letter indicates no significant difference (*p* > 0.05), while different letters indicate significant difference (*p* < 0.05).

Na(+)/K(+)-ATPase is an enzyme mainly located on the membranes of tissue cells and organelles. It plays a crucial role in maintaining the transmembrane ion concentration gradient. Therefore analyzing the intracellular ATPase activity can provide insights into the impact of bacteriostatic agents on the energy metabolism of bacterial cells (Li et al., 2018). As shown in Figure 7, after 6-h culture, the control group exhibited the highest ATPase activity. In contrast, the quercetin treatment group experienced a notable decrease in ATPase activity. Furthermore, as the quercetin addition increased, the ATPase activity showed a significant decrease. It is evident that quercetin can disrupt the normal life activities of *A. acidoterrestris*. These findings imply that quercetin could compromise the integrity of the *A. acidoterrestris* cell membrane, resulting in protein leakage, such as ATPase, a significant contributing factor to the decrease in ATPase activity (Hughes et al., 2021). The decrease in ATPase activity might be attributed to the inhibitory effect of quercetin on *A. acidoterrestris*. Plaper et al. (2003) discovered that quercetin can impede the reproduction of *E. coli* by inhibiting ATPase activity and DNA transcription. Wu et al. (2008) demonstrated that quercetin specifically binds to the enzyme responsible for catalyzing the assembly of d-Ala-d-Ala peptidoglycan precursors, which interacts with the ATP binding region, leading to decreased enzyme activity. Consequently, this mechanism inhibits the enzyme activity of *Helicobacter pylori* and *E. coli*.

## 4. Conclusion

In this study, the antibacterial mechanism of quercetin against *A. acidoterrestris* was investigated. The findings indicated that quercetin exhibited a significant antibacterial effect against *A. acidoterrestris*, showing a minimum inhibitory concentration (MIC) of 100 ug/mL and a minimum bactericidal concentration (MBC) of 400 ug/mL. The SEM results revealed that quercetin induced cell membrane damage. The surface hydrophobicity experiments demonstrated that quercetin increased the surface hydrophobicity of *A. acidoterrestris*, with hydrophobicity intensifying over time. The FDA staining results demonstrated that quercetin could disrupt the cell membrane of *A. acidoterrestris* and increase the cell membrane permeability. The PI staining results indicated that quercetin could induce cell damage by altering the cell membrane permeability of *A. acidoterrestris*, leading to the efflux of nucleic acid substances from the cells. The determination of protein content in the culture medium, bacterial protein content, and Na(+)/K(+)-ATPase activity showed that quercetin could reduce the intracellular protein levels, impede protein expression, and inhibit ATPase synthesis, ultimately triggering apoptosis. In summary, quercetin has the potential to serve as a natural bacteriostatic agent in fruit juice processing, effectively inhibiting the normal growth of *A. acidoterrestris*.

## Data availability statement

The original contributions presented in the study are included in the article/supplementary material, further inquiries can be directed to the corresponding authors.

## Author contributions

XL: Conceptualization, Funding acquisition, Software, Writing – original draft. CT: Data curation, Methodology, Software, Validation, Writing – original draft. YL: Conceptualization, Writing – original draft, Writing – review & editing. JS: Investigation, Project administration, Resources, Writing – review & editing. RZ: Data curation, Investigation, Writing – review & editing. JR: Data curation, Visualization, Writing – review & editing. LJ: Investigation, Validation, Writing – review & editing. JH: Formal analysis, Writing – review & editing. JL: Formal analysis, Writing – review & editing.
